# κ-Opioid receptor stimulation reduces palmitate-induced apoptosis via Akt/eNOS signaling pathway

**DOI:** 10.1186/s12944-019-0989-4

**Published:** 2019-02-14

**Authors:** Yan Cui, Na Feng, Xiaoming Gu, Feng Fu, Jun Li, Haitao Guo, Yali Liu, Shumiao Zhang, Juan Li, Yuanbo Wang, Min Jia, Lu Yang, Fuyang Zhang, Yuemin Wang, Rong Fan, Jianming Pei

**Affiliations:** 1 Department of Nursing, Medical College of Xi’an Peihua University, Xi’an, 710125 Shaanxi Province China; 2 Department of Physiology and Pathophysiology, National Key Discipline of Cell Biology, Fourth Military Medical University, No. 169 West Changle Road, Xi’an, 710032 Shaanxi Province China

**Keywords:** κ-Opioid receptor, Palmitate, Apoptosis, Akt, eNOS

## Abstract

**Background:**

This study was designed to test the hypothesis that κ-opioid receptor (κ-OR) stimulation reduces palmitate-induced HUVECs apoptosis and to investigate its mechanisms.

**Methods:**

HUVECs were subjected to sodium palmitate, apoptosis and cell viability were determined, HUVECs were treated with specific inhibitors to PI3K, Akt, eNOS and siRNAs targeting κ-OR and Akt. Groups were divided as follows: the control group, the sodium palmitate group, the sodium palmitate+U50,488H (a selective κ-OR agonist) group and the sodium palmitate+U50,488H + nor-BNI (a selective κ-OR antagonist) group.

**Results:**

Treatment with sodium palmitate significantly reduced cell viability and increased apoptosis rate which were significantly alleviated by pretreatment with U50,488H, the effect of U50,488H was abolished by nor-BNI. Phosphorylation of Akt and eNOS, as well as NO production were attenuated and accompanied by an increased expression of caspase 3 when HUVECs were subjected to sodium palmitate, and all these changes were restored by pretreatment with U50,488H, the effects of U50,488H were abolished by nor-BNI, and specific inhibitors to PI3K, Akt, eNOS, respectively. SiRNAs targeting κ-OR or Akt abolished the effects of U50,488H on phosphorylation of Akt and eNOS as well as the expressions of caspase 3, Bax and Bcl-2. SiRNAs targeting Akt elicited no effect on the expression of κ-OR.

**Conclusion:**

This study provides the evidence for the first time that κ-OR stimulation possesses anti-palmitate-induced apoptosis effect, which is mediated by PI3K/Akt/eNOS signaling pathway.

## Background

Cardiovascular disease is an important health risk in recent years. As the major regulator of vascular homeostasis, endothelium plays a vital role in the process of atherosclerosis and other related diseases. Endothelium is not only a physical boundary but an active endocrine organ that produces multiple bioactive substances and exerts a wide range of homeostatic function [1]. Endothelium dysfunction is associated with most forms of cardiovascular disease and is thought to play a vital role in the development of atherosclerosis, which remains a leading cause of mortality and morbidity in industrialized societies [2]. Hyperlipidemia is a metabolic syndrome that caused by abnormal increase in blood lipid level, which lead to high risk rate of cardiovascular disease. In the early stage of hyperlipidemia, accumulation and oxidation of low-density lipoprotein cholesterol (LDL-C) give rise to endothelial dysfunction, which is a crucial step leading to atherosclerosis [3]. Therefore, approaches beneficial to the endothelium protection in hyperlipidemia will show a potential in slowing down the progress of atherosclerosis.

An important risk factor in the pathogenesis of atherosclerosis is increased free fatty acids (FFAs) in serum and it is related to an increase in LDL, which has close relationship with the generation of reactive oxygen species (ROS) in endothelium [4]. Overproduction of ROS causes the suppression of Akt/eNOS signaling pathway, reduction in NO production, disturbance of the Bax/Bcl-2 family proteins and the following activation of caspase-3. Thus, it causes activation of the downstream apoptosis protease in the caspase cascade [5]. Palmitate accounts for about 30% of total plasma FFAs. It is reported to be the most common saturated fatty acid that increases in the circulation of diabetic subjects and causes insulin resistance in type 2 diabetes (T2DM) [6, 7]. It has been proved that palmitate is involved in the development of endothelial dysfunction by increasing apoptotic cell death in microvascular and macrovascular endothelial cells through the over-generation of intracellular ROS [8, 9]. Moreover, it has been reported that palmitate-induced endothelial apoptosis at least partly results from mitochondrial dysfunction [10].

In contrast to apoptosis-related signaling pathways, PI3K/Akt/eNOS signaling is of great importance in maintaining the cell survival. PI3K activates its downstream effector Akt through phosphorylation on threonine 308 and on serine 473. The activation of Akt is considered to mediate cell survival in endothelial cells. Akt also causes the production of nitric oxide (NO) by the activation of endothelial nitric oxide synthase (eNOS) [11, 12]. Evidence suggests that the PI3K/Akt/eNOS pathway shows an important role in inhibiting ROS-induced endothelial damage by scavenging superoxide anion, which in turn prevents superoxide anion from forming hydrogen peroxide [5, 13]. Previous studies reported that excessive ox-LDL leads to dephosphorization of Akt/eNOS in a dose and time-dependent fashion in cultured umbilical vein endothelial cells [14]. Other studies in ApoE−/− mouse and STZ-induced diabetes model have also proved that suppression of PI3K/Akt/eNOS pathway and reduction in NO production leads to endothelial dysfunction [5, 7].

In our previous studies it has been demonstrated that considerable κ-opioid receptor (κ-OR) expression exists in vascular endothelium [7]. Stimulation of κ-OR with U50,488H directly dilates vessel in an NO-dependent manner [15]. It also attenuates the elevation in pulmonary artery pressure in rats with hypoxic pulmonary hypertension [16]. U50,488H effectively preserves eNOS activity in HPH rats as well as HUVECs under hypoxic condition, protects pulmonary artery endothelium through antioxidate/nitrative effect and anti-apoptotic effect [15]. We have also found that U50,488H administered immediately prior to reperfusion increases Akt phosphorylation through a PI3K-dependent mechanism and reduces postischemic myocardial apoptosis [17]. Thus, the present study was designed to determine whether κ-OR stimulation with U50,488H protects HUVECs against apoptosis under palmitate treatment and its underlying mechanisms.

## Material and methods

### Cell culture and treatment

The use of human umbilical vein endothelial cell lines (HUVECs) was reviewed and approved by the Ethical Committee of Fourth Military Medical University. HUVECs were purchased from ScienCell Research Laboratories (San Diego, CA). Cells were grown in EGM-2 BulletKit (CC-3162 Lonza) in a 5% CO_2_ incubator. Cells were used within passage 6 after primary culture. HUVECs were incubated with sodium palmitate (450 μmol/L in complete medium) for 48 h to mimic hyperlipidemia condition [18]. U50,488H and nor-BNI were bought from Tocris Bioscience (Bristol, UK). U50,488H was given at a concentration of 70 μmol/L 20 min before sodium palmitate treatment. Nor-BNI was given at a concentration of 10 μmol/L 30 min before sodium palmitate treatment. PI3K inhibitors LY-294002 (20 μmol/L, Sigma), Akt inhibitor MK-2206-2HCl (0.2 μmol/L, Sigma), eNOS inhibitor L-NAME (100 μmol/L, Sigma) were given 30 min before sodium palmitate treatment. All of these reagents were present at the time of treatment of HUVECs with sodium palmitate. The siRNAs targeting human Akt (genepharma, Suzhou, China), human κ-OR (genepharma, Suzhou, China) as well as control siRNA (nontargeting siRNA; genepharma, Suzhou, China) were transfected to HUVECs using the siRNA-Mate transfection reagent (genepharma, Suzhou, China) 12 h before sodium palmitate treatment. At indicated times, HUVECs were harvested. The effectiveness of all chemical inhibitors and siRNAs on HUVECs has been tested in our previous study [19].

### Cell counting kit 8 assay

Cell counting kit 8 (CCK-8) from Dojindo Molecular Technologies, Inc., Kumamoto, Japan was used to measure the cell viability. Briefly, HUVECs were seeded at the density of about 10,000 per well in 96-well microplates, incubated at 37 °C for 24 h. After treated according to different groups mentioned above, 10 μL of CCK-8 solution and 100 μL DMEM was added to each well and incubated at 37 °C for 2 h. The optical density was detected at a wavelength of 450 nm by microplate reader (Model 680, Bio-Rad Laboratories, Hercules, CA, USA). Cell viability was counted following manufacturer’s protocol. The cell viability of the control group was assumed to be 100%.

### Flow cytometry analysis

Cells from different groups were trypsinized and resuspended with cold PBS. An Annexin V-fluorescein isothiocyanate (FITC) apoptosis kit (BD Biosciences, Franklin Lakes, NJ, USA) was used to detect phosphatidylserine externalization as an index of apoptosis. The cells were washed and incubated for 15 min at room temperature in the presence of Annexin V labeled with FITC and propidium iodide (PI). 10,000 cells were loaded and excited at 488 nm, and emission was measured at 530 and 584 nm to assess FITC and PI fluorescence, respectively. Analysis was conducted with BD FACS Calibur flow cytometer (BD Biosciences, Franklin Lakes, NJ, USA). The number of gated cells was plotted on a dot plot with reference to both Annexin V and PI staining.

### Western-blot analysis

Cell lysates were prepared using Radio Immunoprecipitation Assay (RIPA) buffer (Beyotime Biotechnology, China). The protein sample was quantified with the BCA protein assay kit (Beyotime Biotechnology, China). Equal amounts of protein were electrophoresed on a 10% SDS-polyacrylamide gel and electrophoretically transferred to a polyvinylidene difluoride membrane (PALL). After blocking with 5% bovine serum albumin in Tris-buffered saline at room temperature for 1 h, the membranes were incubated with antibody against Akt/phosphorylated Akt (Cell Signaling Technology, Danvers, MA), eNOS/phosphorylated eNOS (BD Bioscience Laboratories, San Jose, CA), κ-OR (GENETEX), Caspase3 (PROTEINTECH), Bax (PROTEINTECH) and Bcl-2 (PROTEINTECH) overnight at 4 °C. Then, the membranes were washed with PBS and incubated with horseradish peroxidase-conjugated IgG antibody for 1 h at room temperature. β-Actin (CWbio, China) was selected as the loading control. The immunoblotting was detected using an enhanced chemiluminescence detection kit (Millipore, Billerica, MA) with ChemiDocXRS system (Bio-Rad Laboratory, Hercules, CA) system. The blot densities were analyzed with Quantity One Software (Bio-Rad Laboratory, Hercules, CA).

### Determination of medium NO content

Total nitric oxide content (NO_x_) in culture medium was determined by measuring the concentration of nitrite, a stable metabolite of nitric oxide, through a modified Griess reaction method. Briefly, medium was taken and mixed with modified Griess reagent according to manufacturer’s protocol (Beyotime Biotechnology, China). The concentration of the resultant chromophore was spectrophotometrically determined at 540 nm.

### Statistical analysis and artwork creation

Data were presented as mean ± SEM. All data were analyzed with either t-test (two group) or ANOVA (three or more groups). After analysis by either t-test (two group) or ANOVA, the Bonferroni correction was conducted for post hoc t-tests. *P*<0.05 was considered to be statistically significant. Graphpad Prism 5 was used to create all artworks.

## Results

### U50,488H attenuated palmitate-induced apoptosis and increased HUVECs viability

**Fig. 1:**
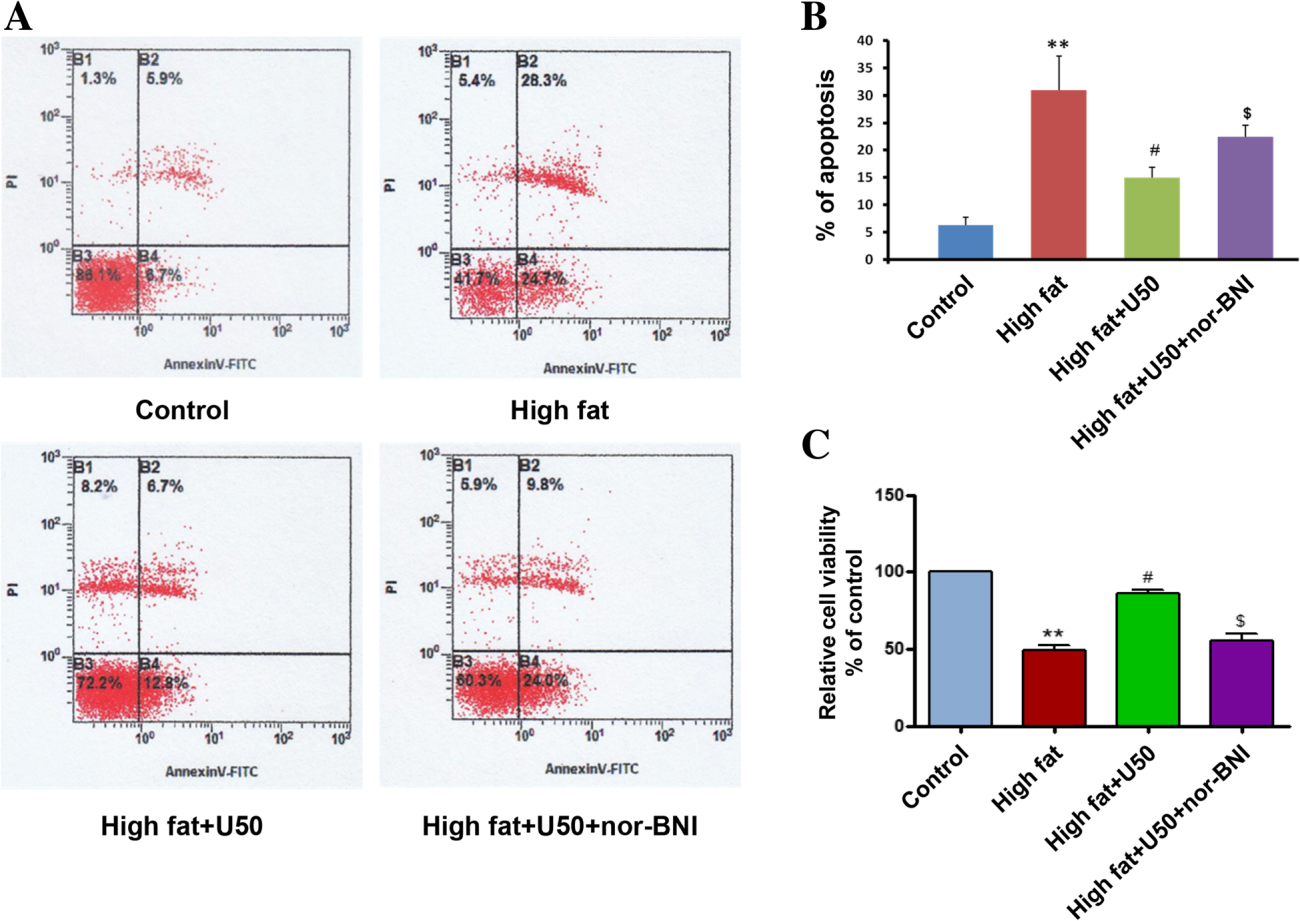
U50,488H ameliorated high fat-induced HUVEC apoptosis. HUVECs were exposed to vehicle or sodium palmitate (450 μmol/L) for 48 h, with or without U50,488H (70 μmol/L) and nor-BNI (10 μmol/L) treatment. **a**-**b** The apoptosis of HUVECs was assayed and quantified by Annexin V-FITC flow cytometry. **c** The cell viability of HUVECs was evaluated by CCK-8 assay. Data were presented as mean ± SEM. *n* = 6. High fat: sodium palmitate, U50: U50,488H, nor-BNI, nor-binaltorphimine. ^**^P < 0.01 vs. Control, ^#^*P* < 0.05 vs. High fat, ^$^*P* < 0.05 vs. High fat +U50

As shown in Fig. 1, after treatment of cells with sodium palmitate for 48 h, apoptosis was prominently increased as increased signal from Annexin V-FITC (Fig. 1a, b), cell survival rate was significantly decreased according to CCK-8 assay (Fig. 1c). U50,488H, a selective κ-OR agonist, significantly reduced apoptosis rate (Fig. 1a, b), and improved the cell viability (Fig. 1c). The effects of U50,488H were blocked by nor-BNI, a selective κ-OR antagonist (Fig. 1b, c). These data indicated that κ-OR stimulation exerts an anti-apoptosis effect in HUVECs subjected to sodium palmitate.

### U50,488H activated Akt/eNOS signaling pathway and enhanced NO production in HUVECs

**Fig. 2:**
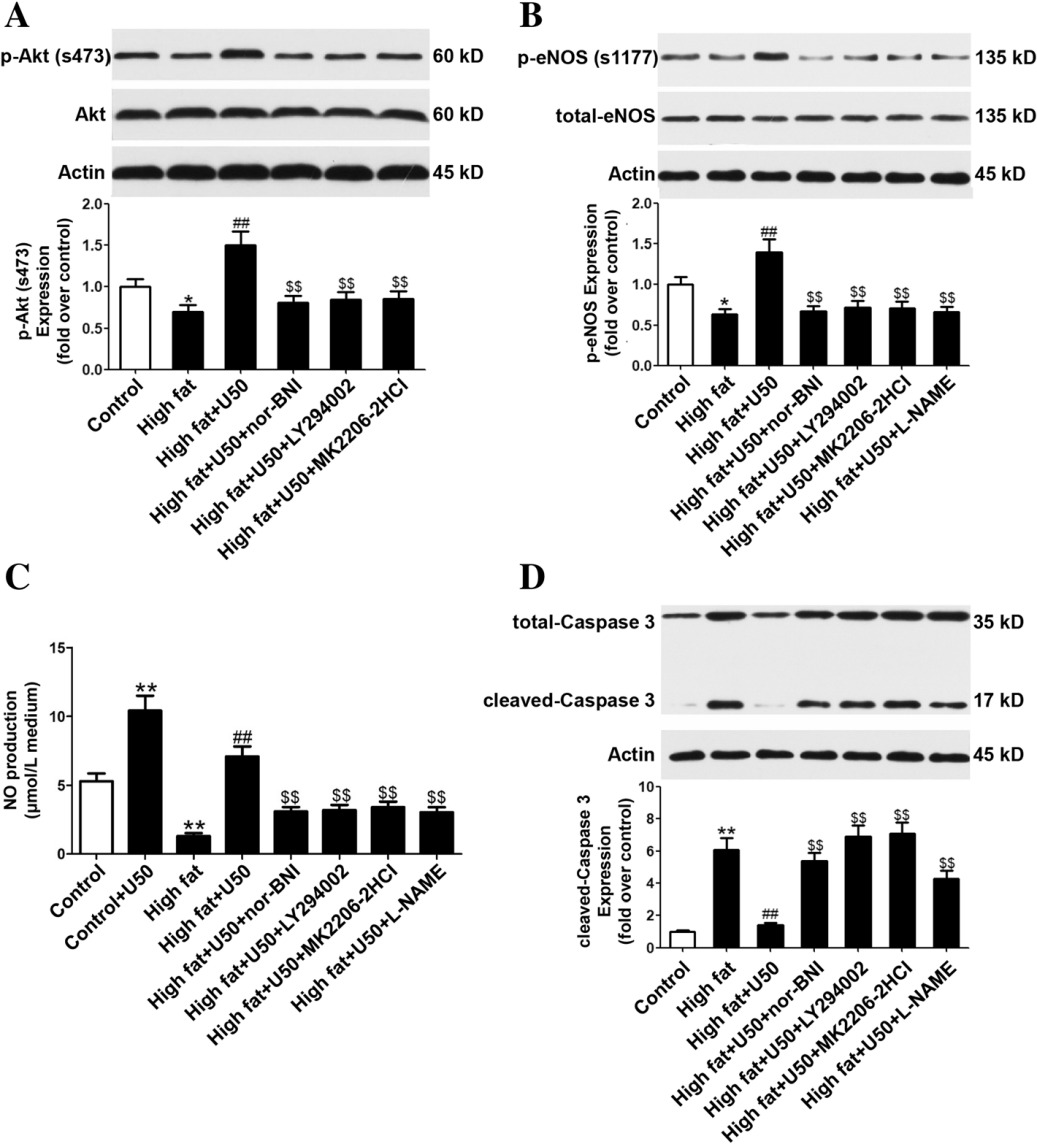
U50,488H activated Akt/eNOS pathway and enhanced NO production. HUVECs were treated with vehicle, palmitate (450 μmol/L), U50,488H (70 μmol/L), Nor-BNI (10 μmol/L), LY-294002 (20 μmol/L), MK-2206-2HCl (0.2 μmol/L), and L-NAME (100 μmol/L) for 48 h. **a**-**b** The expression and phosphorylation of Akt and eNOS were determined by Western blot. **c** The NO production was assayed as methods described. **d** The expression of caspase-3 was determined by Western blot. Values are mean ± SEM. *n* = 6. High fat: sodium palmitate, U50: U50,488H, nor-BNI, nor-binaltorphimine. LY294002, MK2206-HCl, L-NAME were specific inhibitors to PI3K, Akt and eNOS, respectively. ^*^P < 0.05 vs. Control, ^##^*P* < 0.01 vs. High fat, ^$$^*P* < 0.01 vs. High fat +U50

To investigate the mechanism involved in the anti-apoptotic effect of U50,488H, expression and phosphorylation of Akt/eNOS were determined by Western-blot. As illustrated in Fig. 2, treatment with sodium palmitate greatly decreased Akt/eNOS phosphorylation in HUVECs. Pretreatment with U50,488H significantly restored Akt/eNOS phosphorylation. The effect of U50,488H was abolished by nor-BNI, PI3K inhibitor (LY294002), Akt inhibitor (MK-2206-2HCl) and eNOS inhibitor (L-NAME), respectively (Fig. 2a, b). These data indicated that U50,488H activates Akt/eNOS signaling pathway in a κ-OR dependent manner.

At the same time, NO production in HUVECs was detected and it was significantly reduced after sodium palmitate treatment. Pretreatment with U50,488H significantly restored NO production and its effect was abolished by nor-BNI, PI3K inhibitor (LY294002), Akt inhibitor (MK-2206-2HCl) and eNOS inhibitor (L-NAME), respectively (Fig. 2c). These data indicated that U50,488H enhances NO production in HUVECs in a κ-OR dependent manner and through PI3K/Akt/eNOS signaling pathway.

In order to further clarify the relation between κ-OR-mediated Akt/eNOS signaling pathway and anti-apoptosis effect, the expression of caspase 3 in HUVECs was determined. It was found that caspase 3 was significantly increased after sodium palmitate treatment. Pretreatment with U50,488H significantly attenuated cleaved caspase 3 and its effect was abolished by nor-BNI, PI3K inhibitor (LY294002), Akt inhibitor (MK-2206-2HCl) and eNOS inhibitor (L-NAME), respectively (Fig. 2d). These data suggested that the anti-apoptotic effect of U50,488H is probably mediated by PI3K/Akt/eNOS signaling pathway in HUVECs subjected to sodium palmitate.

### U50,488H attenuated palmitate-induced HUVECs apoptosis through PI3K/Akt/eNOS signaling pathway

**Fig. 3:**
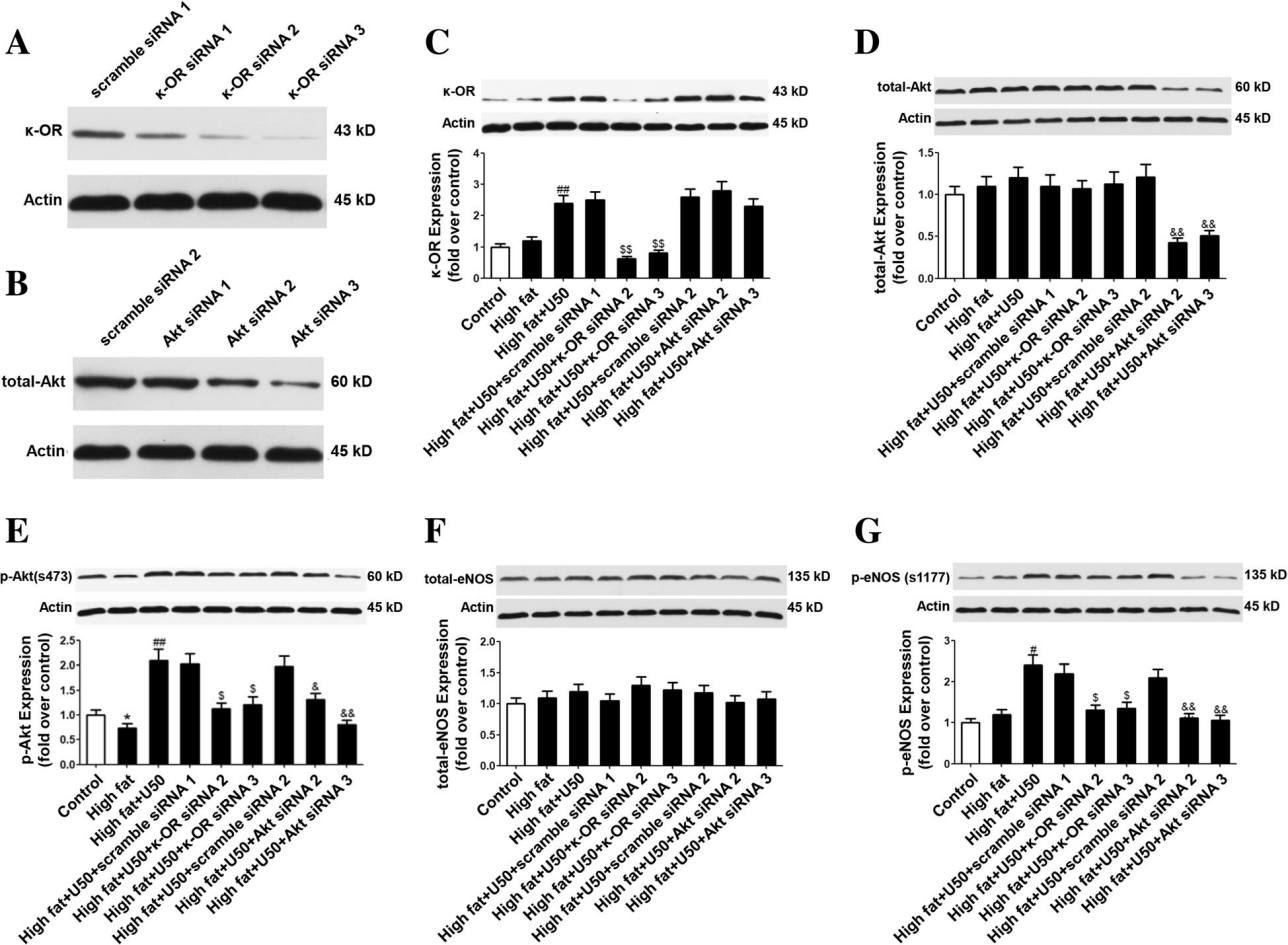
U50,488H inhibited high fat-induced HUVECs apoptosis in a PI3K/Akt/eNOS dependent manner. Cultured HUVECs were transfected with scramble, κ-OR, Akt siRNA for 12 h. **a**-**b** After the transfection, the expression of κ-OR and Akt were determined by Western blot. **c**-**g** In scramble, κ-OR or Akt siRNA-transfected HUVECs, sodium palmitate (450 μmol/L) and U50,488H (70 μmol/L) were added for 48 h. The expression of κ-OR, Akt, phosphorylated Akt, eNOS and phosphorylated eNOS were determined by Western blot. Values are means±SEM. *n* = 4. High fat: sodium palmitate, U50: U50,488H, ^*^P < 0.05, ^**^P < 0.01 vs. Control, ^#^*P* < 0.05, ^##^*P* < 0.01 vs. High fat, ^$^*P* < 0.05, ^$$^*P* < 0.01 vs. High fat +U50 + scramble siRNA1, ^&^*P* < 0.05, ^&&^*P* < 0.01 vs. High fat +U50 + scramble siRNA2

To confirm the role of key molecules in the endothelial protective effect of U50,488H, sodium palmitate-impaired HUVECs were treated with siRNAs targeting κ-OR and Akt. The silence effects of siRNAs were tested and exhibited (Fig. 3a, b). According to the silence effect, κ-OR siRNA 2 and 3 or Akt siRNA 2 and 3 were adopted in the further study. The up-regulation of the expression of κ-OR by U50,488H was suppressed by κ-OR siRNA whereas it was not affected by Akt siRNA (Fig. 3c). Although sodium palmitate, U50,488H and κ-OR siRNAs in medium did not affect total Akt/eNOS expression in HUVECs (Fig. 3d, f), total expression of Akt was significantly decreased in the Akt siRNAs-treatment group (Fig. 3d). Sodium palmitate significantly reduced Akt/eNOS phosphorylation (Fig. 3e, g) whereas it was greatly up-regulated by U50,488H. SiRNAs targeting κ-OR or Akt abolished the effects of U50,488H on Akt/eNOS phosphorylation (Fig. 3e, g).

**Fig. 4:**
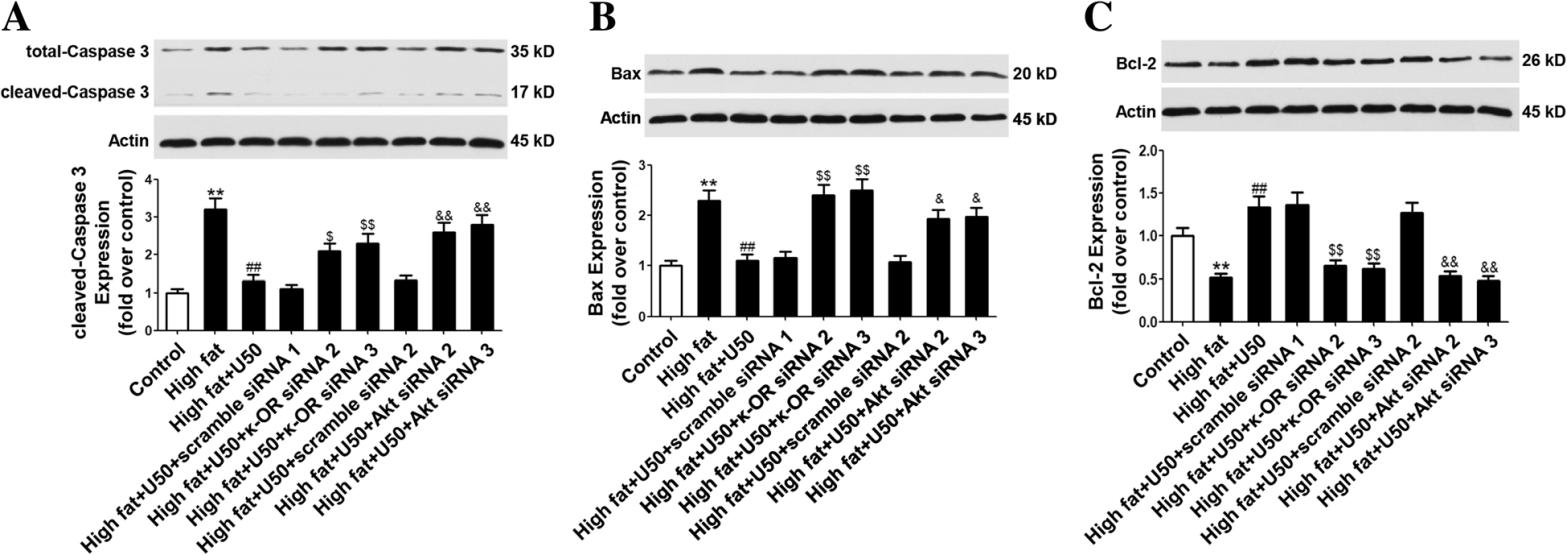
U50,488H regulated pro- and anti-apoptotic protein expression through PI3K/Akt/eNOS signaling pathway. Cultured HUVECs were transfected with scramble, κ-OR, Akt siRNA for 12 h. In scramble, κ-OR or Akt siRNA-transfected HUVECs, sodium palmitate (450 μmol/L) and U50,488H (70 μmol/L) were added for 48 h. **a**-**c** The expressions of caspase-3, Bax, and Bcl-2 were determined by Western blot. Values are means±SEM. *n* = 4. High fat: sodium palmitate, U50: U50,488H. ^**^P < 0.01 vs. Control, ^##^*P* < 0.01 vs. High fat, ^$$^*P* < 0.01 vs. High fat +U50 + scramble siRNA1, ^&^*P* < 0.05, ^&&^*P* < 0.01 vs. High fat +U50 + scramble siRNA2

In order to further demonstrate the relation between κ-OR-mediated Akt/eNOS signaling pathway and anti-apoptosis effect, apoptosis proteins such as caspase 3, Bax and Bcl-2 were determined. Treatment with sodium palmitate significantly increased the expression of caspase 3, Bax and reduced the expression of Bcl-2(Fig. 4a, b, c), whereas all these changes was restored by pretreatment with U50,488H. SiRNAs targeting κ-OR or Akt abolished the effects of U50,488H on the expression of caspase 3, Bax and Bcl-2 (Fig. 4a, b, c). Results above suggested that the anti-apoptotic effect of U50,488H is mediated by κ-OR activation and the PI3K/Akt/eNOS signaling pathway.

## Discussion

**Fig. 5:**
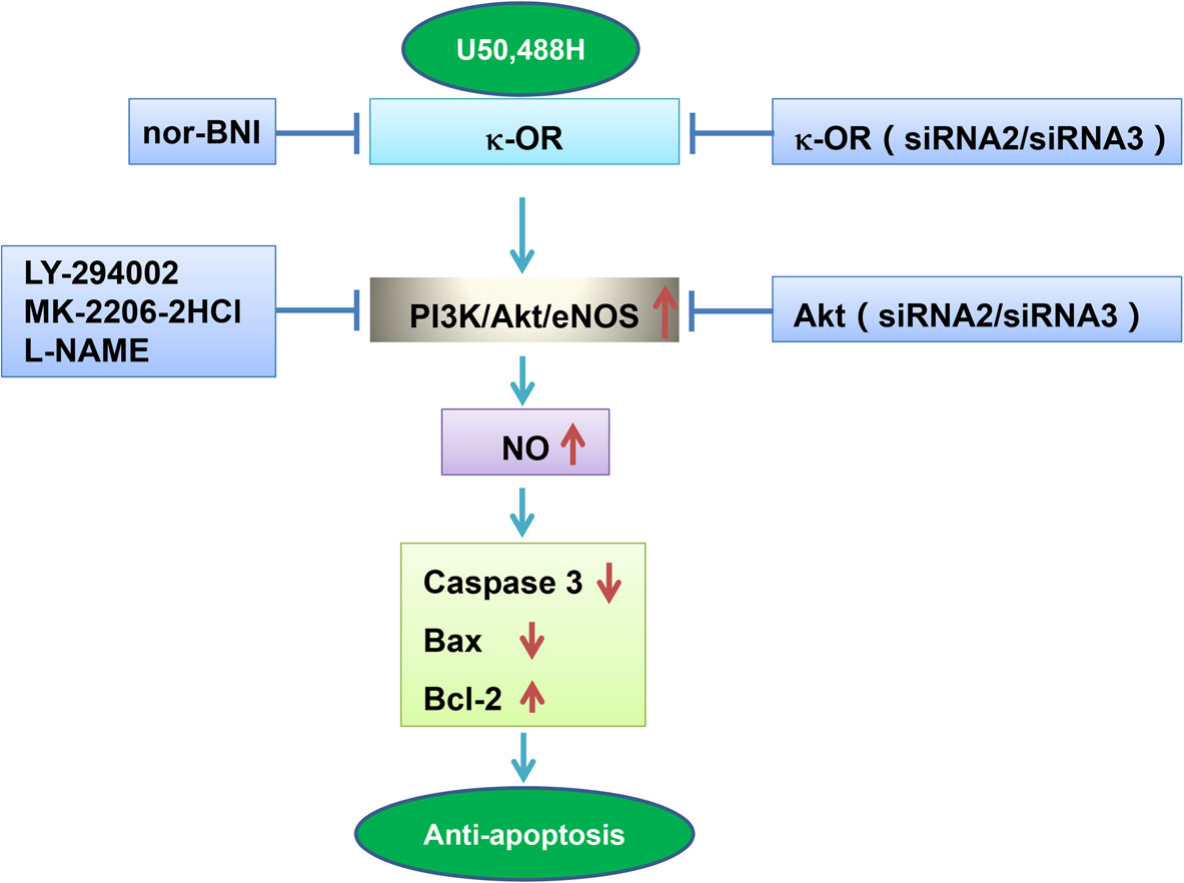
Graphic abstract. In this figure, it shows that apoptosis occurred when the HUVECs were subjected to sodium palmitate, κ-OR stimulation with U50,488H significantly attenuated this apoptosis via PI3K/Akt/eNOS signaling pathway. κ-OR, κ-opioid receptor; U50,488H, a selectiveκ-OR agonist; nor-BNI, a selective κ-OR antagonist; LY-294002, an inhibitor of PI3K; MK-2206-2HCl, an inhibitor of Akt; L-NAME, an inhibitor of eNOS; NO, nitric oxide; Caspase 3 and BAX are apoptosis proteins, Bcl-2 is an anti-apoptosis protein

As an independent risk factor of atherosclerosis, hyperlipidemia induces a series of molecular events including ox-LDL accumulation, ROS overproduction, eNOS uncoupling, and finally leading to an increased endothelium apoptosis. Previous studies demonstrated that the activation of PI3K/Akt pathway, restoring eNOS activity and suppressing of oxidation/nitration possesses endothelial protection ability [19]. In the present study, we proved for the first time that preventative treatment with U50,488H showed significant effect to ameliorate HUVECs apoptosis caused by sodium palmitate through the activation of κ-OR and PI3K/Akt/eNOS pathway. This conclusion is based on the following observations: 1) Flow cytometry and CCK-8 analysis proved an anti-apoptotic effect of κ-OR stimulation with U50,488H. 2) U50,488H treatment restored Akt/eNOS phosphorylation and NO production which was suppressed by palmitate treatment. The effects of U50,488H were abolished by chemical inhibitors to PI3K (LY294002), Akt (MK-2206-2HCl) and eNOS (L-NAME), respectively. 3) U50,488H treatment suppressed palmitate induced activation of caspase 3 and expression of Bax. It also increased the expression of anti-apoptotic molecule Bcl-2. 4) The effect of U50,488H on apoptosis related molecules was blocked by chemically inhibition on PI3k/Akt/eNOS signaling pathway and siRNAs targeting κ-OR and Akt. 5) All the effects of U50,488H were abolished by nor-BNI. Our findings suggest that κ-OR stimulation plays an important role in the regulation of endothelial apoptosis induced by sodium palmitate (Fig. 5).

Palmitate is reported to be the most common saturated fatty acid that increases in the circulation of diabetic subjects and causes insulin resistance in T2DM [6]. In vitro, palmitate has also been proved to promote HUVECs apoptosis through excessive production of ROS and mitochondrial dysfunction [18]. In the present study we confirmed that sodium palmitate promotes HUVECs apoptosis. This effect was supported by multiple evidences, including expression of Bcl-2/Bax, cleaved caspase-3 protein expression and flow cytometry analysis on Annexin V-FITC/PI. The effect of sodium palmitate was suppressed significantly by the pre-treatment with U50,488H, and the effect of U50,488H was blocked by nor-BNI. We also found that sodium palmitate suppressed Akt and eNOS phosphorylation, which could be restored by pre-treatment with U50,488H, all effects of U50,488H was also blocked by nor-BNI. These phenomena indicate that the anti-apoptotic effect of U50,488H is κ-OR mediated and Akt/eNOS signaling pathway is involved. In addition, in the present study, it was found that κ-OR protein expression was up-regulated by U50,488H, which provided the basis for the action of U50,488H, further study is needed to elucidate the underlying mechanism.

In order to uncover the signaling mechanism of κ-OR, inhibition experiment on PI3K/Akt/eNOS signaling pathway was conducted with chemically inhibitors and siRNAs targeting κ-OR and Akt. The inhibitory effects of U50,488H on caspase 3 were significantly blocked by chemical inhibitors to PI3K (LY294002), Akt (MK-2206-2HCl) and eNOS (L-NAME). This result is consistent with our previous in vivo reports in rats treated with chronic hypoxia [15] and chronic high fat diet^16^. These results confirm that the anti-apoptotic effect of U50,488H is probably mediated by PI3K/Akt/eNOS signaling pathway. Since eNOS activation and in turn enhancing NO production play a critical role in endothelial function, we further determined Akt/eNOS phosphorylation and NO production, it was found a loss of Akt/eNOS phosphorylation as well as a reduction in NO production occurred in palmitate-treated HUVECs, which was restored by preventive treatment with U50,488H. The effects of U50,488H on Akt/eNOS phosphorylation and NO production were significantly blocked by chemical inhibitors to κ-OR (nor-BNI), PI3K (LY294002), Akt (MK-2206-2HCl) and eNOS (L-NAME), respectively. Furthermore, SiRNAs targeting κ-OR or Akt abolished the effects of U50,488H on Akt and eNOS phosphorylation. Results above suggested that the anti-apoptotic effect of U50,488H is mediated by κ-OR activation and the PI3K/Akt/eNOS signaling pathway.

Caspases, a group of vastly well-preserved, cysteine- dependent and aspartate-specific proteases, play a vital role in the regulation and execution of apoptosis. It was wildly accepted as signal of apoptosis. There are two types of caspases: initiator caspases as caspase 2, 8, 9 and 10, and effector caspases including caspase 3, 6 and 7. Initiator caspases activate effector caspases by cleave inactive pro-forms of them. Effector caspases in turn cleave other protein substrates at aspartate residues within the cell. Both death receptors-related and cell stress-related apoptosis pathways are related to caspase. The two pathways congregate at caspase-3 activation [20]. When the full-length pro-caspase 3 (32kD) is activated, it is cleaved to form two mature subunits, p17 (17kD) and p12 (12kD). The level of the cleaved caspase 3 represents its activation. The caspase pathway is also a well-identified downstream target for PI3K/Akt/eNOS. One mechanism by which PI3K/Akt/eNOS regulates cell survival involving in the S-nitrosylation of cysteine 163 in the active center of the subunit p17 of caspase 3, which attenuates of its activity [21]. Our study showed that treatment with sodium palmitate caused an increase in cleaved caspase 3, which means an increased apoptosis. Treatment with U50,488H alleviated this alteration. The effect of U50,488H was blocked by pretreatment with nor-BNI. The caspase 3 suppressing effect of U50,488H could also be significantly blocked by both siRNAs targeting κ-OR or Akt and chemical inhibitors to κ-OR(nor-BNI), PI3K (LY294002), Akt (MK-2206-2HCl) and eNOS (L-NAME). These results prove an anti-apoptotic effect of U50,488H and its relationship with PI3K/Akt/eNOS signaling pathway.

Bcl-2 and Bax are also closely related to apoptosis [22]. Bcl-2 is mainly contained as an integral mitochondrial membrane protein that forms heterodimers with Bax to prevent mitochondrial changes in apoptosis [23]. Our results showed that U50,488H significantly reduced the increase in the expression of Bax caused by palmitate. It also significantly restored the expression of Bcl-2, an anti-apoptotic protein. These effects of U50,488H could also be significantly blocked by siRNAs targeting κ-OR or Akt. Our findings indicated that the upregulation of Bcl-2 and downregulation of Bax ratio also contribute to the anti-apoptotic effect of U50,488H.

There are still some limitations in our study. First, our experiments have showed that κ-OR stimulation with U50,488H exerts anti-apoptotic effect via PI3K/Akt/eNOS signaling pathway, while how κ-OR stimulation induces the activation of PI3K is still unclear, which may need further investigation. Second, palmitate-induced endothelial cell apoptosis was also mediated by an increasing ROS generation, and AMPK signaling activation suppressed palmitate-induced apoptosis [7]. Whether ROS and AMPK signaling are involved in U50,488H-induced anti-apoptotic effect is not known, which warrants further study. Despite these limitations, we believe that this study has provided an important new information about the protective effect of κ-OR stimulation against palmitate-induced endothelial cell apoptosis.

In conclusion, the present study provides evidence for the first time that κ-OR stimulation inhibits palmitate induced HUVECs apoptosis through activation of PI3K/Akt/eNOS signaling pathway and beneficial regulation of Bax, caspase 3 and Bcl2. Our work provides new insight for the preventative effects of κ-OR stimulation in endothelial cells, which may give a pharmacological basis for the clinical application of U50,488H or similar compounds for treatment of hyperlipidemic disease, which is related to endothelial cell apoptosis.

## Abbreviations

Akt: Serine/threonine kinase
eNOS: Endothelial nitric oxide synthase
FFAs: Free fatty acids
HPH: Pulmonary arterial hypertension
HUVECs: Human umbilical vein endothelial cell lines
LDL: Low-density lipoprotein
NO: Nitric oxide
nor-BNI: nor-Binaltorphimine
ox-LDL: Oxidized low-density lipoprotein
PI3K: Phosphatidylinositol 3-hydroxy kinase
ROS: Reactive oxygen species
T2DM: Type 2 diabetes
κ-OR: κ-opioid receptor
